# Hot-Melt Extruded Amorphous Solid Dispersion for Solubility, Stability, and Bioavailability Enhancement of Telmisartan

**DOI:** 10.3390/ph14010073

**Published:** 2021-01-18

**Authors:** Bhupendra Raj Giri, Jaewook Kwon, Anh Q. Vo, Ajinkya M. Bhagurkar, Suresh Bandari, Dong Wuk Kim

**Affiliations:** 1 College of Pharmacy, Research Institute of Pharmaceutical Sciences, Vessel-Organ Interaction Research Center (VOICE, MRC), BK21 FOUR Community-Based Intelligent Novel Drug Discovery Education Unit, Kyungpook National University, Daegu 41566, Korea; bhupendra@knu.ac.kr (B.R.G.); kjw11156@naver.com (J.K.); 2 Department of Pharmaceutics and Drug Delivery, School of Pharmacy, The University of Mississippi, University, MS 38677, USA; anhvq@hup.edu.vn (A.Q.V.); ajinkya.bhagurkar@allergan.com (A.M.B.); sbandari@olemiss.edu (S.B.); 3 Department of Physical Chemistry and Physics, Hanoi University of Pharmacy, Hanoi 100000, Vietnam

**Keywords:** telmisartan, pH-modifier, solid dispersion, hot-melt extrusion (HME), solubility, bioavailability, stability

## Abstract

Telmisartan (TEL, an antihypertensive drug) belongs to Class II of the Biopharmaceutical Classification System (BCS) because of its poor aqueous solubility. In this study, we enhanced the solubility, bioavailability, and stability of TEL through the fabrication of TEL-loaded pH-modulated solid dispersion (TEL pH_M_-SD) using hot-melt extrusion (HME) technology. We prepared different TEL pH_M_-SD formulations by varying the ratio of the drug (TEL, 10–60% *w*/*w*), the hydrophilic polymer (Soluplus^®^, 30–90% *w/w*), and pH-modifier (sodium carbonate, 0–10% *w*/*w*). More so, the tablets prepared from an optimized formulation (F8) showed a strikingly improved in vitro dissolution profile (~30-fold) compared to the free drug tablets. The conversion of crystalline TEL to its amorphous state is observed through solid-state characterizations. During the stability study, F8 tablets had a better stability profile compared to the commercial product with F8, showing higher drug content, low moisture content, and negligible physical changes. Moreover, compared to the TEL powder, in vivo pharmacokinetic studies in rats showed superior pharmacokinetic parameters, with maximum serum concentration (C_max_) and area under the drug concentration–time curve (AUC_0_–_∞_) of the TEL pH_M_-SD formulation increasing by 6.61- and 5.37-fold, respectively. Collectively, the results from the current study showed that the inclusion of a hydrophilic polymer, pH modulator, and the amorphization of crystalline drugs in solid dispersion prepared by HME can be an effective strategy to improve the solubility and bioavailability of hydrophobic drugs without compromising the drug’s physical stability.

## 1. Introduction

Hypertension is a condition with a rise in blood pressure and is considered a global health issue that affects 25% of adults [1]. Generally, Telmisartan (TEL) is a potent, long-lasting angiotensin-II type 1 (AT1) receptor blocker used for treating and controlling essential hypertension and preventing renal impairment caused by diabetes and cardiovascular diseases [2,3]. TEL has a higher binding affinity and the longest half-life (t_1/2_ ~ 24 h) compared to commercially available Sartans such as Losartan, Valsartan, and Irbesartan, making it possible for once-daily dosing to induce effective blood pressure control throughout the day, thereby improving the overall quality of patients’ life [4,5].

TEL is categorized as a BCS Class II molecule because of its low aqueous solubility (0.09 µg/mL) with dissolution rate-limited absorption. Additionally, TEL is highly ionizable (pKa 4.45 ± 0.09) and shows pH-dependent solubility behavior, i.e., sparingly soluble in strongly acidic media but readily soluble at strong alkaline conditions [6]. Though TEL is in the limelight as an effective antihypertensive drug, its highly pH-dependent and poor solubility features cause inconsistent absorption and insignificant bioavailability (~43%), leading to its suboptimal therapeutic activity [7,8]. Moreover, commercially available TEL products, particularly MICARDIS^®^, are loaded with a high concentration of hygroscopic excipients, specifically strong alkalizers such as sodium hydroxide that makes the product unstable when exposed to environmental conditions. Therefore, a drug delivery technique that can enhance the solubility, dissolution, and pharmacokinetic features of TEL, providing product stability under environmental storage conditions, is highly desired. 

Lately, numerous formulation strategies have been developed to address the limitations of low aqueous solubility and poor bioavailability associated with BCS Class II compounds. Some of these techniques are the use of prodrug formulations [9], self-emulsifying systems [10], salt formulations [11], size reduction techniques (micro/nano-size preparations) [12], oral lipid-based formulations (SMEDDS/SNEDDS) [13], cyclodextrin complexations [14], solid dispersion (SD) [15,16,17], etc. SD is one of the most widely accepted and elementary methods that involves the dispersion of an active pharmaceutical ingredient (API) in solubilized, amorphous, or microcrystalline forms in an inert hydrophilic carrier/s. The entrapment of drugs within hydrophilic carriers induces better wettability, drug amorphization, and particle size reduction, which are the main mechanisms by which an amorphous solid dispersion (ASD) system improves drug dissolution [18]. In recent years, hot-melt extrusion (HME) has been one of the most preferred techniques used to fabricate SD for improving the solubility of hydrophobic molecules. Observably, compared to conventional methods for preparing SD, such as kneading, melt evaporation, spray drying, and freeze-drying, HME has many advantages such as simplicity and high efficiency and solvent and dust-free processing, making it environment-friendly; its single continuous manufacturing that does not require additional drying and fewer processing steps makes it feasible for large-scale manufacturing, thereby providing economic advantages [19,20]. Moreover, the intense mixing of the drug and carrier performed at an elevated temperature and the shear force allow homogeneous dispersion of the drug and carrier, which ultimately increases the possibility of drug–carrier interaction at the molecular level, forming a solid solution. Additionally, this causes transformation of the crystalline drug to an amorphous form and stems maximum-specific surface area and higher saturation solubility that induce enhanced drug solubility and bioavailability [20].

Furthermore, several formulation strategies have been used to overcome the poor solubility barrier associated with TEL such as size reduction, i.e., nanoparticle formulations [21], TEL aminoclay complex formulation [22], TEL cyclodextrin nanocomposites [23], TEL–Chitosan SD [24], TEL-SD system prepared via supercritical antisolvent [25], melt quenching [26,27], and solvent evaporation techniques [28,29,30]. Most of these studies focused on the use of pH-modifiers and the amorphization of crystalline drugs as a method to overcome the hydrophobic nature of TEL. Consequently, the solubility and dissolution rate and oral bioavailability were strikingly enhanced. However, the physical stability of the pH-modulated ASD system during storage was affected because of the inclusion of pH-modifiers and the amorphization of crystalline drugs [31,32]. Despite the fact that the solubilization and pH modulations by pH-modifiers have a positive significance on the dissolution and pharmacokinetic performance of the ASD, it can cause negative consequences by absorbing moisture from the environment, thereby lowering the T_g_ of the ASD system, consequently, making the formulation unstable and less effective. Additionally, the thermal denaturation of carriers during HME processing can affect drug dissolution performance collectively, causing formulation failure. Therefore, for effective product development, it is critical to increase the solubility and maintain the stability of the ASD system. However, investigation of the solubility, stability, and overall pharmacokinetic performance of the TEL-loaded pH-modulated solid dispersion system prepared via HME has not been fully explored.

Therefore, we fabricated and evaluated the TEL-loaded pH_M_-SD system using HME technology to improve solubility, dissolution rate, and stability, thus enhancing the pharmacokinetics of TEL. This will provide greater insight into the influence of pH-modifiers on the solubility and stability of a TEL-loaded solid dispersion formulation. Furthermore, this study provides a better understanding of HME technology in developing soluble and stable oral pharmaceuticals with poorly water-soluble and ionizable drugs.

## 2. Results and Discussion

### 2.1. Solubility of TEL in Different pH

As a preliminary study, we investigated the solubility of TEL through aqueous solubility tests in various buffers of pH 1.2, 4.0, 6.8, and 10.0, and distilled water. The solubility of TEL was highest at strong alkaline conditions i.e., 2557.7 µg/mL at pH 10 followed by the acidic environment (97.2 µg/mL at pH 1.2) (Table 1). As predicted, the aqueous solubility of the drug was poor in distilled water (4.4 µg/mL). Overall, the results suggested that TEL shows a strong pH-dependent solubility characteristic with high aqueous solubility in basic or acidic environments, but very limited solubility in neutral conditions. Hence, alkalizers were chosen, particularly as the pH-modifiers instead of acidifiers, for preparing SD. These solubility results conform to those in the literature, where the solubility of TEL was shown to be highly pH-dependent and practically insoluble between pH 3 and 9 [6].

### 2.2. Formulation and Optimization of TEL-Loaded ASD

A careful selection of carrier/s is a prerequisite for developing an SD system as the aqueous solubility of the carrier influences the degree of solubilization and dissolution because of its high solubility and diffusivity values. Furthermore, the use of a highly miscible carrier reduces the melting point of the active without causing significant drug degradation, thus facilitating the HME thermal processing [33]. Moreover, to prepare a stable SD system, the chosen carrier/s should stabilize and decrease the molecular kinetics of the drug within the drug–carrier matrix system, preventing recrystallization of the amorphous drug, thereby increasing solubility [20,26,34].

**Figure 1:**
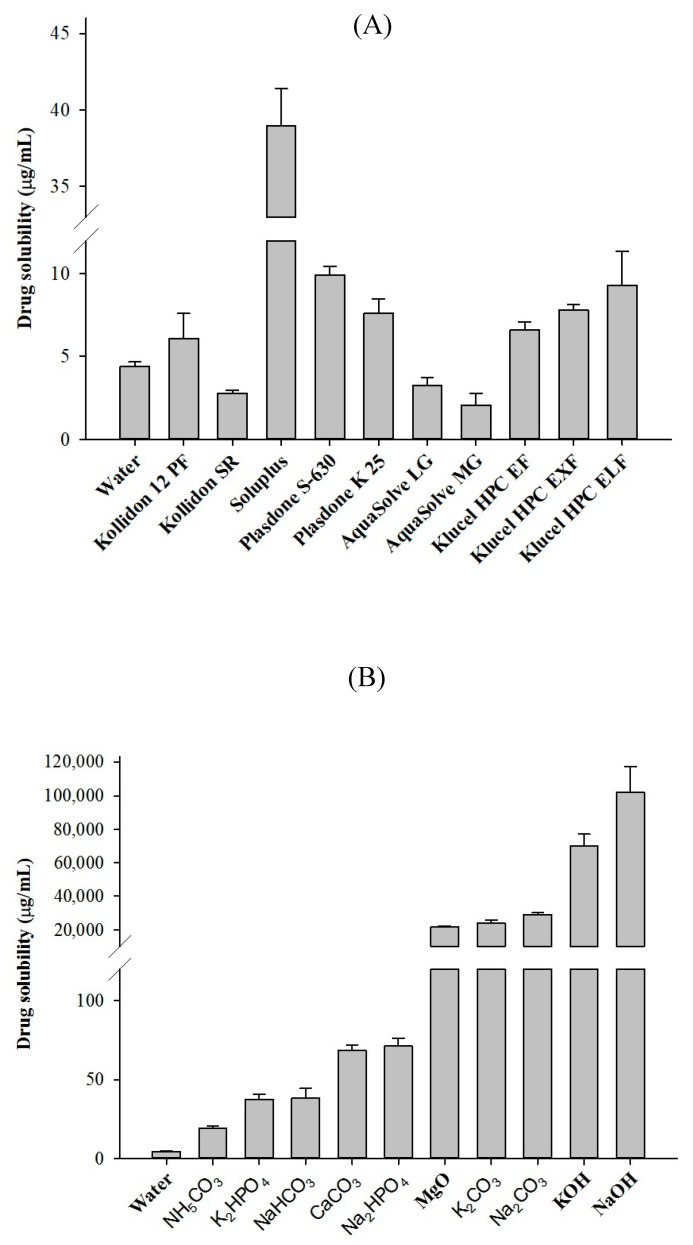
Aqueous solubility of TEL in different: (**A**) polymer solutions (1% *w*/*v*) and (**B**) alkalizer solutions (1% *w*/*v*). Each value represents the mean ± SD (*n* = 3).

To select the most suitable polymeric carrier, the aqueous solubility of the drug in 1% (*w/v*) of various hydrophilic polymers and alkalizing agents was assessed. Among the hydrophilic polymers, Soluplus^®^ showed the highest solubility (38.97 µg/mL), whereas AquaSolve MG with 2.05 µg/mL showed the lowest drug solubility (Figure 1A). Soluplus^®^ is a new type of thermoplastic polymer specifically developed for use in the HME process and has several advantages [35,36,37]. Particularly, the amorphous nature and low glass transition (T_g_) temperature of Soluplus^®^ make it easily extrudable via HME, therefore eliminating the need to add a plasticizer in the formulation [33,38]. Thus, Soluplus^®^ was chosen as a hydrophilic polymer to prepare the hot-melt extruded TEL-loaded pH_M_-SD system. 

Similarly, careful selection of a pH-modifier is another key consideration in the development of microenvironmental pH-modulated formulation for drugs showing pH-dependent solubility [32]. The inclusion of a pH-modifier into the ASD system probably induces some molecular interactions among functional groups of drug and pH-modifiers to form a stable supersaturation state and might prevent drug recrystallization. Studies have shown that the microenvironmental pH may vary depending on the type of pH-modifiers used in the formulation, and transitions of microenvironmental pH cause differences in drug solubility and further dissolution characteristics [6,39,40]. Conversely, the inclusion of pH-modifiers in the development of SD formulations probably causes poor chemical stability and manufacturability [32], inducing formulation failure. Therefore, careful selection of suitable pH-modifiers is vital for a stable pH_M_-SD system. Among several investigated alkalizers, sodium hydroxide (NaOH), potassium hydroxide (KOH), and sodium carbonate (Na_2_CO_3_), respectively, showed the highest aqueous solubility of TEL (Figure 1B). Presumably, this is caused by a strong alkalizer that increases the microenvironment pH, thereby allowing maximum drug release into the alkaline environment. Since NaOH and KOH have stability issues as both are highly hygroscopic in nature, Na_2_CO_3_ was specifically chosen as an alkylating agent in this study.

**Figure 2:**
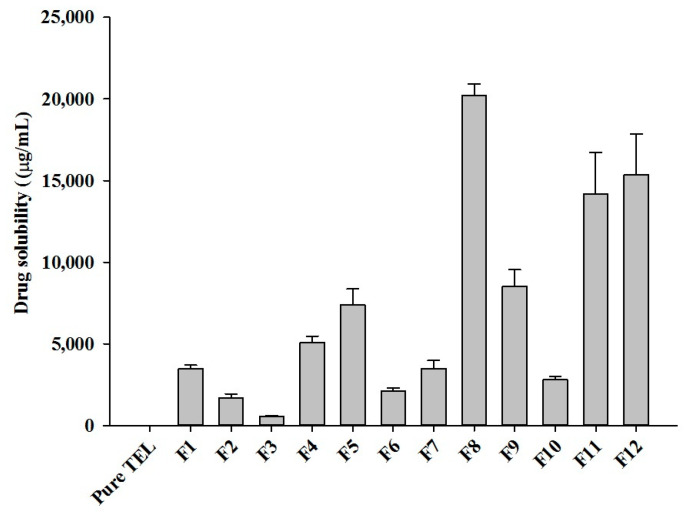
Aqueous solubility of different HME TEL-loaded pH-modulated solid dispersion prepared by varying drug/carrier ratios. Each value represents the mean ± SD (*n* = 3).

Thus, TEL-loaded pH_M_-SDs were prepared using Soluplus^®^ as a hydrophilic polymer and Na_2_CO_3_ as a pH-modulating agent using a lab-scale hot-melt extruder. During the preliminary experiments, HME conditions such as barrel temperature, screw speed, feed rate, and die temperature were optimized. Twelve SD formulations with varying drug-carrier ratios were prepared and subjected to an aqueous solubility test to find the best drug–carriers combination. Among the prepared formulations, the eighth formulation (F8) comprising drug/Soluplus^®^/Na_2_CO_3_ at 40/55/5 (% *w*/*w*) showed the highest drug solubilizing effect (20,208.33 µg/mL) followed by F12 and F11, respectively (Figure 2). Collectively, the optimized formulation, i.e., F8, showed a notable improvement in drug aqueous solubility, approximately by 5000-fold compared to the pure TEL powder. However, in the absence of a microenvironment pH-modifier (formulations without alkalizer: F1, F4, F7, and F10), drug solubility was relatively lower and considered as incomplete drug release (Figure 2). The result reveals the significance of incorporating a pH-modifier in a ternary SD system and presents a potential alternative to overcome the challenges with BCS Class II drugs having pH-dependent solubility characteristics. Nevertheless, despite further addition of carriers, drug solubility was not increased once the maximum solubility was achieved with F8. This might possibly be due to the formation of a supersaturated (concentrated) layer of the solution in the vicinity of drug particles and higher polymer leaching from the formulation, which could have hindered the TEL release in aqueous media [15]. These results further support the rationale of screening the drug-carrier ratio and suggest that adding a high concentration of carriers does not guarantee the highest drug solubility in SD formulation. Based on these observations, F8 was chosen as an optimized pH_M_-SD formulation for our further studies.

### 2.3. Evaluation of TEL-Loaded ASD

**Figure 3:**
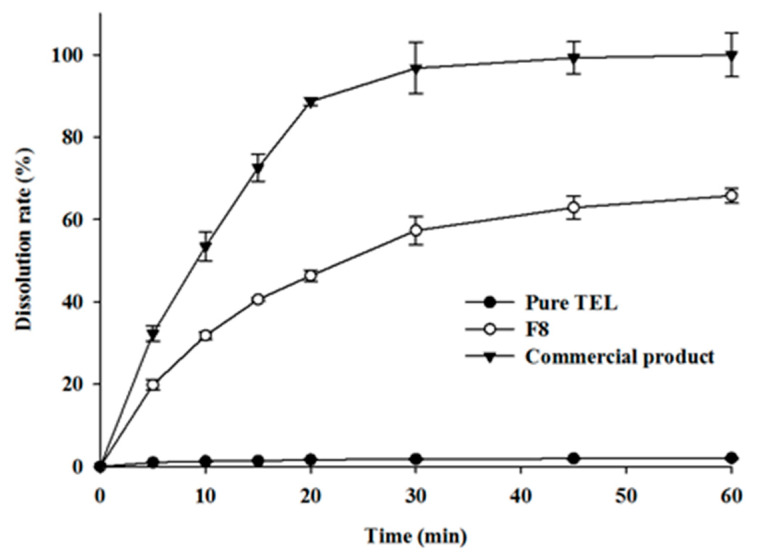
Dissolution profiles of drugs from tablets containing pure TEL powder, TEL-loaded pH-modulated solid dispersion tablet (F8), and a commercial product (MICARDIS^®^). Each value represents the mean ± SD (*n* = 3).

To understand the influence of alkalizers on dissolution kinetics, an in vitro dissolution test was carried out using pure TEL, commercial tablet (MICARDIS^®^), and compared to the in-house-developed TEL-loaded pH_M_-SD tablet (F8 tablet) made via direct compression of the optimized formulation. The dissolution curves of pure TEL, F8, and commercial product are shown in Figure 3. The release profiles are represented by the percentage of drug released vs. time (min). The percentage of drug release from the pure TEL, F8, and commercial product was 2.07 ± 0.18%, 65.78 ± 1.8%, and 99.9 ± 5.3%, respectively. By 45 min, both the F8 and commercial tablets had dissolved to their utmost state, displaying a plateau afterwards. 

The in vitro dissolution rate of free TEL tablets was very limited because of its poor aqueous solubility (Figure 3). Conversely, F8 tablets showed an approximately 30-fold higher cumulative drug release profile compared to the free drug tablets. This might be attributed to the inclusion of SD containing hydrophilic polymers and alkalizer (Na_2_CO_3_), which accelerates drug release upon contact with the dissolution media. The alkalizer acts as a pH modulator to raise the microenvironmental pH of media surrounding drug molecules up to an alkaline condition that creates a favorable environment for the maximum drug release, resulting in higher TEL release. The microenvironmental pH is the pH of the saturated solution measured near the solid formulations such as the diffusion layer. Additionally, Na_2_CO_3_ might act as a precipitation inhibitor during drug dissolution, thereby maintaining the stable supersaturation state, thus allowing higher TEL release in the dissolution media [41]. More so, the molecular dispersion of the drug within the hydrophilic polymer (Soluplus^®^) in the SD system improves the wettability and solubilization of TEL [33,34] that synergistically induce increased TEL release. Tran et al. (2010) [40] observed that when the dissolution media penetrate the tablets, they release pH-modifiers enveloping the hydrophobic drug and maintain an optimal microenvironmental pH surrounding the drug particles, inducing improved drug release. Additionally, molecular interaction between the functional groups of drug and carriers (i.e., hydrogen bonds or electrostatic), particle size reduction (micronization of the particles during HME), and transformation of the crystalline drug into its amorphous state (as suggested by DSC and PXRD data), collectively contribute to an improved drug release from the F8 formulation. Evidently, the drug in the amorphous state shows better solubility than its crystalline form since no or low energy must break up the crystal lattice during the dissolution of the amorphous drug [25,34]. Thus, these results suggest that the TEL-loaded pH_M_-SD system showed an improved dissolution profile for poorly water-soluble TEL. 

Observably, the dissolution kinetics of F8 tablets was lower compared to the commercial tablets (Figure 3), possibly caused by the inclusion of numerous excipients in the commercial tablet, which probably induced higher drug release. Specifically, sodium hydroxide, (NaOH, a strong alkalizer), povidone (K25, widely used as binder and tablet disintegrant), and meglumine (a solubilizing agent) which could have resulted in higher solubility, therefore, enhanced the dissolution profile of the commercial formulation compared to the F8 tablets. Addition of a few excipients such as strong alkalizer (NaOH), super disintegrant, or solubilizing agents may increase the release kinetics of TEL from the F8 tablets as reported in the prior literature [30,42]. However, the study aims to address the stability issue observed with the marketed formulation (MICARDIS^®^) and meanwhile, enhance the solubility and bioavailability of poorly soluble TEL. Therefore, a compromise was made between drug dissolution and stability, which led to a ~30-fold increase in drug release as compared to pure TEL and significantly improved stability compared to the commercial product.

### 2.4. Solid-State Characterization

#### 2.4.1. Scanning Electron Microscopy (SEM)

**Figure 4:**
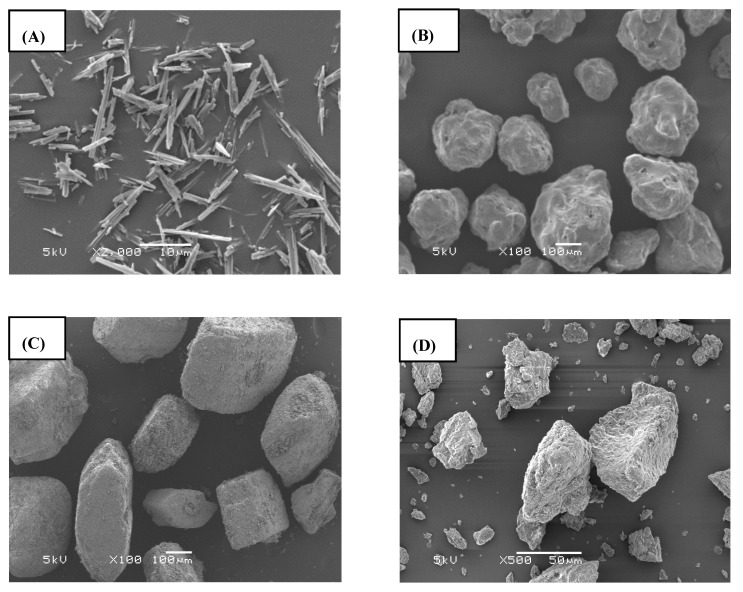
Scanning electron micrographs: (**A**) TEL powder; (**B**) Soluplus ^®^; (**C**) Sodium carbonate (SC); and (**D**) TEL-loaded pH-modulated solid dispersion (F8).

Physicochemical characterization is a useful technique widely used to investigate the solid-state properties of drugs, carriers, and formulations because of its adequate resolution and high magnification. The morphological characteristics of free TEL, Soluplus^®^, Na_2_CO_3_, and F8 were examined using SEM. The SEM images of free TEL showed long and needle-shaped particles (Figure 4A), whereas notable changes in surface topography were observed with HME (F8), having discrete and slightly coarse surface particles (Figure 4D). Additionally, the SEM images of Soluplus^®^ and Na_2_CO_3_ (Figure 4B,C) showed larger, rough, and irregular-shaped particles. Generally, only with the SEM micrographs, it is difficult to confirm the crystallinity of the samples. However, the absence of crystallinity is possibly expected in F8 due to the disappearance of free needle-shaped structures, suggesting that the drug possibly adsorbed and/or dispersed within the carriers. Furthermore, we used DSC and PXRD to verify the amorphous state of the TEL-loaded pH_M_–SD system.

#### 2.4.2. Differential Scanning Calorimetry (DSC)

**Figure 5:**
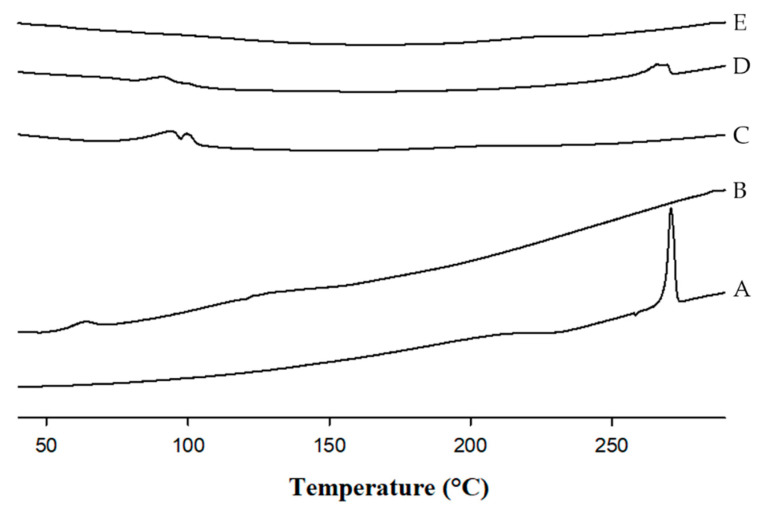
DSC thermograms of (**A**) TEL powder; (**B**) Soluplus^®^; (**C**) Sodium carbonate (SC); (**D**) physical mixture (PM); and (**E**) TML-loaded pH-modulated solid dispersion (F8).

DSC is a destructive technique basically used in pharmaceutical technology to better understand the physicochemical properties of a drug, PM, or final formulation. The transition from the glassy state to the rubbery state involves an enthalpy relaxation and the resulting peaks are detected in the DSC. In this study, the presence of a crystalline structure was suspected because TEL powder showed a single sharp endothermic peak at roughly 270 °C (Figure 5A), similar to its melting point [37,43]. Characteristic sharp peaks for Soluplus^®^ and SC (Figure 5B,C) were absent; however, a small and broad endothermic peak was observed at approximately 60–70 °C, which corresponds to the T_g_ of Soluplus^®^ [37] and at 90–110 °C, probably caused by the effect of residual moisture present in Na_2_CO_3_. Nevertheless, the physical mixture (PM) showed two broad endothermic peaks both with reduced intensities at approximately 90 and 260 °C, respectively (Figure 5D), indicating the crystallinity. Generally, the presence of amorphous carriers tends to adsorb with the drug in the PM, which lowers the temperature at which the transition occurs (lower T_g_ of TEL in PM) followed by the broadening effect of the peak [15]. 

Conversely, the absence of a characteristic peak was observed in the DSC thermogram of F8 over the entire scanned temperature range (Figure 5E). During the HME processing, the uniform blending of API and carrier matrix is accelerated at elevated temperature under the influence of heat and shear, causing drug molecules to lose their molecular mobility. Additionally, the drug molecules become entrapped within the carriers and “freeze”, inhibiting nucleation and the absence of any crystal structures [34,44]. Hence, the disappearance of the drug melting endotherm might possibly be due to the molecular level mixing of drug and carriers induced due to shear heat and high pressure during HME processing [20], resulting in the alteration of the crystalline TEL into its amorphous form. 

#### 2.4.3. Powder X-ray Diffraction (PXRD)

**Figure 6:**
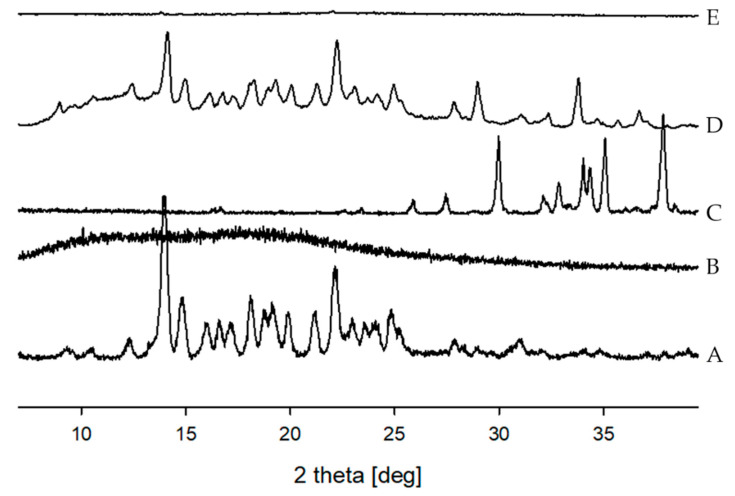
X-ray powder diffraction of (**A**) TEL powder; (**B**) Soluplus^®^; (**C**) Sodium carbonate (SC); (**D**) physical mixture (PM); and (**E**) TEL-loaded pH-modulated solid dispersion (F8).

Additionally, PXRD was used to further confirm the amorphous nature of the formulation. Figure 6 illustrates the PXRD patterns for the TEL powder, Soluplus^®^, Na_2_CO_3_, PM, and F8. The diffraction patterns of pure TEL showed numerous sharp peaks with high intensities largely around 10–25° (*2θ*), implying the highly crystalline nature of TEL (Figure 6A). Because of the amorphous nature of Soluplus^®^, distinct peaks were absent (Figure 6B). More so, the X-ray diffraction patterns of PM showed that most of the characteristic peaks were observed in those of pure TEL and SC XRD patterns (Figure 6D); contrastingly, sharp XRD peaks were absent with F8 (Figure 6E). The absence of PXRD diffraction peaks with F8 confirms that a high concentration of the TEL is dissolved in its solid state, leading to its amorphization, which agrees with the DSC and SEM results. 

The drug molecules with stable crystal lattices suffer from poor solubilization due to its elevated lattice energy. Conversely, the enthalpy, entropy, and free energy of an amorphous system are generally higher than those of the crystalline state [45]; therefore, this excess free energy of amorphous TEL probably aided the higher dissolution kinetics of TEL-loaded pH_M_-SD. Thus, the alteration of the solid-state characteristics of the crystalline drug to its amorphous forms using HME technology offers distinct advantages of simplicity and higher solubility of TEL. 

### 2.5. Stability Studies

The significance of stability should not be neglected in pharmaceutical product development, even though improved solubility of BCS Class II drugs is the prime intention of formulation scientists. The major concern of using an amorphous system is that it lacks physical stability due to its inherent high thermodynamic instability (excess enthalpy and entropy). Consequently, SDs tend to undergo recrystallization upon storage, resulting in conversion to the same poorly soluble crystalline drug, inducing formulation failure [46]. Moreover, TEL is also known to be slightly hygroscopic [47] and fabrication of the SD system of TEL further increases concerns as per the stability of the developed formulation. Thus, it is critical to investigate the stability of the prepared TEL-loaded pH_M_-SD.

**Figure 7:**
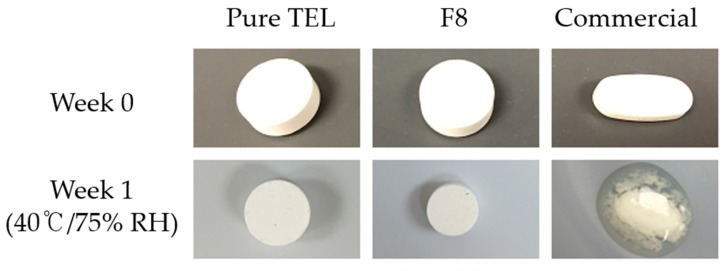
The stability test, the physical appearance of Pure TEL tablet (**left side**); TEL-loaded tablet (F8, **middle**); and Commercial tablet (MICARDIS^®^, **right side**).

For the stability test, F8 and the commercial tablets were exposed in an open state for 4 weeks under accelerated conditions (40 °C/75% RH). Initially, F8 and commercial products did not show any considerable physical differences (Figure 7); however, a week later, the commercial tablet showed notable changes in appearance—watery-like appearance with a small soft cake-like mass in the middle. Conversely, pure TEL and F8 tablets remained unchanged during the same storage conditions. A possible explanation is the inclusion of strong hygroscopic excipients, namely NaOH and povidone K25, used in commercial tablet formulations, showing the hygroscopic property, i.e., they tend to absorb moisture from the environment resulting in deliquescence of the commercial tablet.

**Figure 8:**
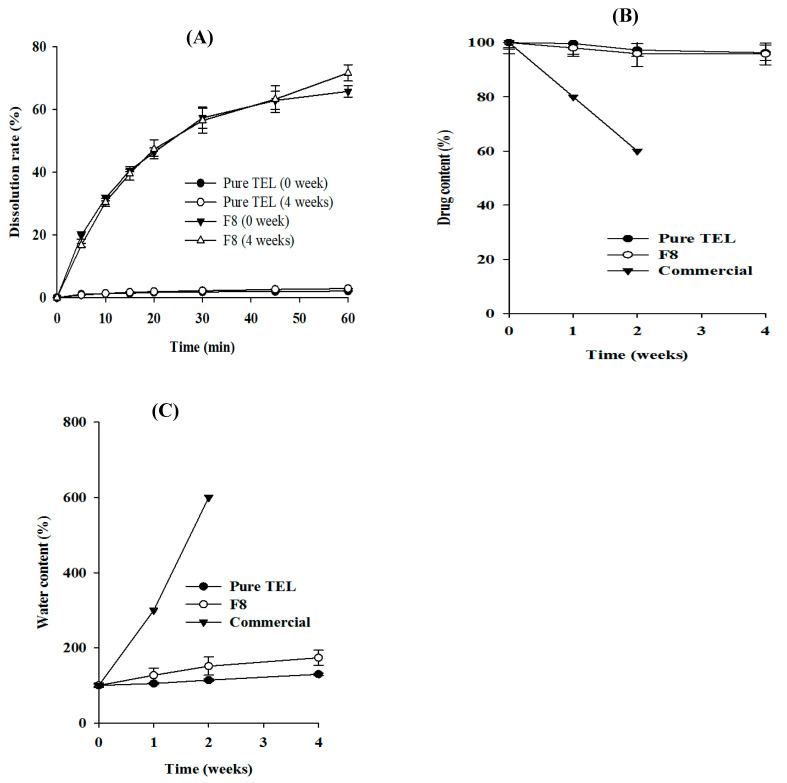
Stability testing. (**A**) In vitro dissolution rate; (**B**) drug content; and (**C**) water content of pure TEL tablet, TEL-loaded solid dispersion tablet (F8), and commercial tablet (MICARDIS^®^) during storage. Each value represents the mean ± SD (*n* = 3).

To investigate the effect of heat and moisture on the dissolution kinetics, an in vitro dissolution study was performed weekly for 4 weeks with pure TEL, F8, and commercial tablets. At the end of the study period, we found that the overall dissolution profile of the TEL and F8 tablets did not have striking differences in their release kinetics compared to their initial drug release profiles (Figure 8A). After a few days of storage, it was difficult to monitor the dissolution profile of the commercial tablet due to its high deliquescent nature that makes it very unstable.

Furthermore, the effect of temperature and moisture on the water and drug content of the F8, pure TEL, and commercial (MICARDIS^®^) tablets were investigated for 4 weeks in accelerated conditions. The initial drug contents of TEL, F8, and commercial tablets were 0.41, 0.34, and 0.36 g, respectively, which were assigned as 100%. After 4 weeks, the calculated drug contents of the TEL and F8 tablets were 96.3 ± 2.3% and 95.8 ± 4.8%, respectively (Figure 8B). Conversely, the drug content of the commercial tablet dropped remarkably, hitting a lower value of roughly 60% (Figure 8B) due to its very unstable characteristics. 

Moreover, initially (0 week), water content in the pure TEL, F8, and commercial tablets was 1.48 ± 0.29%, 2.89 ± 0%, and 2.13 ± 0.57%, respectively (Figure 8C); all were assigned 100%. Four weeks later, the water contents in the pure TEL and F8 tablets were 1.92% (~130%) and 5.0% (~173%), respectively. However, 2 weeks after the study period, the water content in the commercial tablets was significantly higher (~600%) and because of its thick viscous watery-like presence, it was difficult to obtain the water content thereafter.

Collectively, all these stability results suggested that the hygroscopicity of the tablets developed from the TEL-loaded pH_M_-SD was lower and the formulation was more stable compared to the commercial product. However, in order to conclude that the amorphous state was intact even after the storage period, a more thorough analysis was required.

### 2.6. In Vivo Pharmacokinetic Studies

**Figure 9:**
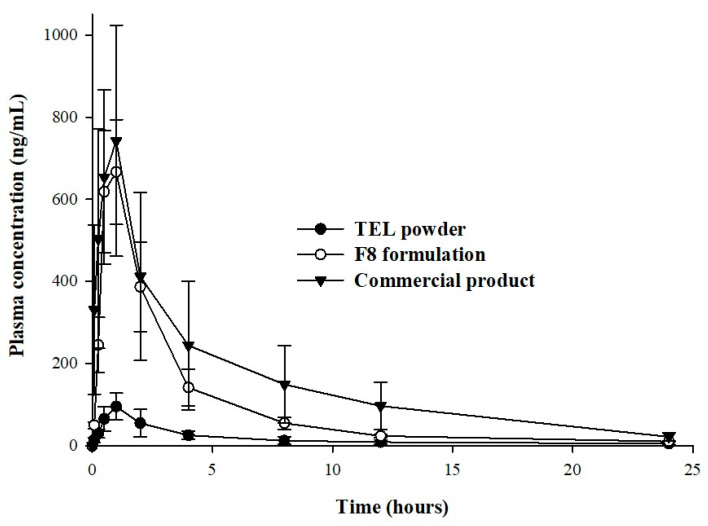
Plasma concentration–time profiles of TEL after oral administration of free drug (TEL), commercial formulation, or prepared amorphous solid dispersion formulation (F8) in rats. Each value represents the mean ± SD (*n* = 6).

The oral bioavailability of the pure TEL powder, commercial formulation, and TEL-loaded pH_M_ ASD (F8) was evaluated in rats (*n* = 6) and the mean plasma concentration–time profiles and pharmacokinetic (PK) parameters are summarized in Table 2. As shown in Figure 9, the plasma concentrations–time curve of untreated TEL increased slowly, giving T_max_ of about 0.83 h, meaning that the pure TEL drug was absorbed slowly, resulting in C_max_ and AUC_0_–_∞_ of 105.46 ± 21.02 ng/mL and 423.69 ± 114.82 h·ng/mL, respectively. Conversely, drug absorption was relatively quick with F8 at all time points, with a brief T_max_ period of 0.75 h and a higher TEL plasma concentration having C_max_ and AUC_0_–_∞_ of 697.51 ± 92.65 ng/mL and 2275.21 ± 776.84 h·ng/mL, respectively. The mean plasma concentration and area under the curve of F8 were increased approximately by 6.61- and 5.37-fold compared to the free TEL drug. Additionally, the in vivo pharmacokinetic parameters, mainly C_max_ and AUC of the F8 formulation, were lower compared to the commercial formulation i.e., 697.51 ± 92.65 vs. 757.27 ± 244.72 ng/mL and 2275.21 ± 776.84 vs. 3425.42 ± 1553.04 h·ng/mL, respectively. This increase in PK results with the commercial formulation is mainly due to the higher drug dissolution (Figure 3), leading to higher in vivo drug absorption from the commercial formulation (Figure 9).

The in vivo results agree with those of the in vitro and suggest that the increased oral bioavailability is the outcome of the improved solubility and dissolution rate of TEL. The total oral bioavailability of the TEL-loaded pH_M_-SD system increased noticeably (~5.37-fold); observably, the therapeutic dose could be lowered by approximately 5.37 times to obtain the same level of pharmacotherapeutic response as that achieved by the unprocessed TEL. Clinically, this approach might offer several other advantages such as improved disease response with reduced drug dose, use of less or low concentration of excipients (generally, a large number of excipients in higher amounts are associated with conventional oral dosage forms), reduced side effects (due to low chemical consumption), improved cost-effectiveness, and overall enhanced patient compliance.

## 3. Materials and Methods

### 3.1. Materials

The United States Pharmacopeia (USP) standard TEL powder was kindly supplied by Hanmi Pharm. Co. (Suwon, Korea). MICARDIS^®^ (40 mg) tablets marketed by Boehringer Ingelheim were purchased from the local market and used as a commercial formulation in this study. Soluplus^®^ was generously gifted from BASF (SE Pharma Ingredients & Services, Ludwigshafen, Germany) and Na_2_CO_3_ was purchased from Duksan Chemical Co. (Ansan, Korea). The drug and carriers used in this study were summarized in Table 3. All other chemicals were used as received. 

### 3.2. Methods

#### 3.2.1. Solubility Study of TEL

The saturation solubility test was performed in various media to investigate the pH-dependent solubility of TEL. Excess TEL powder was placed into microtubes (Eppendorf, Westbury, NY, USA) containing 1 mL of test media: enzyme-free simulated gastric fluid (pH 1.2), acetate buffer (pH 4.0), enzyme-free simulated intestinal fluid (pH 6.8), phosphate buffer (pH 10) adjusted by 1 N NaOH solution, or deionized water. The samples were vortexed and then placed under mechanical stirring at 25 ± 0.5 °C at 100 rpm for 5 days, followed by centrifugation at 10,000× *g* for 10 min. The supernatant was collected, filtered through a 0.45 µm PTFE syringe filter, and then diluted with mobile phase to quantify the TEL concentration using HPLC (Agilent 1260 Infinity, Agilent technologies, Santa Clara, CA, USA). The HPLC system had a pump (Agilent 1260 Quat pump), a Capcell Pak C18 column (Shiseido, 250 × 4.6 mm I.D., 5 μm) maintained at a 35 °C oven temperature. The mobile phase consisted of a 40:60 (*v*/*v*) mixture of potassium dihydrogen phosphate and acetonitrile (pH 3.7) eluted at 1 mL/min and 10 μL volume of injection. The signals were monitored with an Agilent 1260 VWD detector at λ = 296 nm. 

#### 3.2.2. Solubility Screening of Polymers and Alkalizers

In order to select the appropriate solid carriers for SD, various hydrophilic polymer and alkalizers were evaluated for their aqueous solubility with the drug. An excess of TEL powder was added to 1 mL aqueous solution of each polymer or alkalizer and the suspension was vortexed. Then, the samples were mechanically stirred (water bath) for 5 days at 25 °C and 100 rpm. The resulting suspension was centrifuged at 10,000× *g* for 10 min, subsequently filtered using a 0.45 µm PTFE syringe filter. The filtrate was sufficiently diluted with the mobile phase and assessed for TEL concentration by HPLC method, as described above. All experiments were carried out in triplicate (*n* = 3). 

#### 3.2.3. Preparation and Optimization of pH-Modulated Solid Dispersions and Tablets

The polymer that showed the highest aqueous drug solubility and an alkalizing agent (chosen specifically) from the screening result were selected as appropriate carriers. Then, the mixture of drug and carriers was blended roughly 10 min using a V-shell blender and extruded using a co-rotating twin-screw mini HME (Haake MiniLab II, Thermo Fisher Scientific, Karlsruhe, Germany) to obtain the TEL-loaded pH_M_-SDs. To determine the optimum drug-carriers ratio, 12 formulations were prepared by varying the weight ratio of the drug (telmisartan, TEL), polymer (Soluplus^®^, SOL), and alkalizer (sodium carbonate, SC) (Table 4). The HME processing conditions were barrel temperature 150–160 °C, screw speed 100 rpm, and feed rate 3 g/min, respectively. Thus, generated extrudate was milled into a fine powder by means of a laboratory grinder and sieved through a USP #30-mesh to produce a fine dry powder. Furthermore, all the prepared formulations were evaluated for their aqueous solubility test as per the method described above. The formulation showing the highest aqueous drug solubility was chosen as the most appropriate and used for further studies.

We prepared the tablets for dissolution and stability studies using direct compression via a single-punch tablet press machine (MCTMI, GlobePharma Inc., New Brunswick, NJ, USA). Before direct tablet compression, Flowlac 90/Ac-Di-Sol/magnesium stearate was mixed at a weight ratio of 90/9/1. Then, the excipient mixture was introduced to the drug powder (40 mg) or optimized formulation (100 mg). Subsequently, the final powder mixture (200 mg) was manually fed into the die (10 mm) and pressed with a compression force of 500 psi to obtain flat-faced tablets of 200 mg total weight, each containing 40 mg of equivalent TEL. The hardness of the tablets was manually evaluated and compared to the commercial tablets.

#### 3.2.4. In Vitro Dissolution Study 

The drug release profiles of pure TEL tablets, in-house-developed tablets, and the marketed tablets were computed using a USP type II dissolution apparatus (ERWEKA; DT 620, Heusenstamm, Germany). The samples were placed into the dissolution tester filled with 900 mL of distilled water at 37 ± 0.5 °C with a paddle stirring speed of 75 rpm. The powder sample was loaded in a size “0” capsule and the capsules were placed inside sinkers and subjected to dissolution testing. One milliliter of sample was collected at different time intervals (0, 5, 10, 15, 20, 30, and 60 min), and an equivalent volume of fresh medium was subsequently replenished into the vessel to compensate for the media loss. The samples were immediately filtered using a 0.45 µm PTFE syringe filter and sufficiently diluted with mobile phase. After sufficient dilution of the filtrate, the content of TEL was quantified by the HPLC, as detailed above. All experiments were repeated in triplicate.

### 3.3. Solid-State Characterizations

#### 3.3.1. Scanning Electron Microscopy (SEM)

The morphological evaluation of the powder samples was analyzed using an SEM (SU8220, Hitachi, Tokyo, Japan) operated at 5.0 kV. The samples were placed onto metal stubs using a double-sided adhesive carbon tape, placed on a brass specimen holder, and coated with platinum (6 nm/min) using an EMI Teck Ion Sputter system (K 575 K). The images were taken from different points.

#### 3.3.2. Differential Scanning Calorimetry (DSC)

The thermographs of TEL, carriers, and formulation powder were investigated employing a DSC Q20 (TA Instruments; New Castle, DE, USA). All samples weighed to roughly 5–10 mg and were sealed, placed in a pierced aluminum pan, and heated from 50 to 290 °C at a constant heating rate of 10 °C/min under a nitrogen purge gas flow of 50 mL/min. The PM contains the same proportion of drug, polymer, and alkalizer as that of the optimized formulation. 

#### 3.3.3. Powder X-ray Diffraction (PXRD)

XRD curves of the samples were obtained with a D/MAX-2500 XRD instrument (Rigaku, Japan) with Cu-Kα radiation (1.54178 Å, 40 kV, and 40 mA). The samples were scanned at room temperature in 0.05° steps from 5° to 40° (2θ) and scanning speed of 4°/min.

### 3.4. Stability Studies

We investigated the effect of environmental elements such as humidity and temperature on the quality of the in-house-prepared TEL tablets measured as per external morphological changes, in vitro drug release features, moisture content, and drug load, and compared it with the commercial product. Using a thermostatically controlled stability chamber, the pure TEL-loaded tablets, TEL-loaded pH_M_-SD tablets (F8), and commercial tablets were placed in glass vials and stored at an accelerated condition (40 ± 2 °C/75 ± 0.5% RH). The tablets were regularly examined to observe any physical changes. Furthermore, at a known time interval (0, 1, 2, and 4 weeks), 3 tablets were withdrawn from the chamber and the in vitro drug release rate and content were determined as per the method described above. Similarly, for hygroscopic analysis, each tablet sample was subjected to a loss on drying (LOD) test (MB45 moisture analyzer, Ohaus, Switzerland) by heating at 105 °C for 10 min. The percentage of moisture content was determined at 0, 1, 2, 3, and 4 weeks and plotted.

### 3.5. Pharmacokinetic Studies

#### 3.5.1. Animals

For in vivo pharmacokinetic studies, 18 male Sprague-Dawley rats (Samtako Bio Korea, Osan, Korea), 7–8 weeks old and weighing 270 ± 20 g, were used. The protocol approved by the Institutional Animal Care and Use Committee developed at the Kyungpook National University (Permit number: 2019–0054, date of consent: March 01, 2019) was followed for animal experiments. Three groups each containing six rats per cage were kept in a controlled condition of 25 ± 2 °C room temperature and 55 ± 5% RH for 3 days. Prior to the experiment, animals were fasted for 12 h and had free access to tap water. Each individual rat was orally administered a 1 mL aqueous suspension of the TEL powder, commercial formulation, or optimized formulation (F8) at a single dose of 20 mg/kg body weight using oral gavage. After being anesthetized with diethyl ether, approximately 0.25 mL of blood was collected at specified time intervals via the jugular vein and transferred into heparinized microtubes. Blood samples were centrifuged at 13,000× *g* for 10 min at 4 °C to isolate the plasma and stored at −20 °C for further analysis.

#### 3.5.2. In Vivo Plasma Sample Preparation

TEL and Candesartan (as an internal standard, IS) were extracted from plasma by the protein precipitation technique in which 100 μL of ACN was added to 90 μL of plasma sample and 10 μL of Candesartan (200 ng/mL) and vortexed for few minutes. The samples were centrifuged at 13,000× *g* for 10 min and immediately, the supernatant layer was transferred to HPLC vials for quantification. The mobile phase consisted of a 40:60 (*v*/*v*) mixture of potassium dihydrogen phosphate and acetonitrile (pH 3.7) eluted at 1 mL/min and 20 μL of injection volume. The other HPLC conditions were similar to as described earlier. The standard plasma calibration curve was linear (R^2^ = 0.996) across the range of 10–2000 ng/mL.

#### 3.5.3. Statistical Data Analysis

Each point in the plasma concentration–time curve represents a mean of six recordings (*n* = 6) and the data are reported as mean ± SD. The pharmacokinetic parameters of TEL were estimated using non-compartmental analysis with Phoenix^®^ WinNonlin^TM^ (Certara^®^, Princeton, NJ, USA) software. The area under the drug concentration–time curve (AUC_0_–_∞_) was determined from using trapezoidal summations [29,37]. The peak plasma concentration (C_max_) and time to reach the C_max_ (T_max_) were observed from the plasma concentration versus time plots. The statistical analyses were conducted using the unpaired Student’s *t*-test and a difference of *p*-value lower than 0.05 was considered statistically significant.

## 4. Conclusions

In this study, we have successfully developed a microenvironment pH-modulated amorphous SD system of poorly water-soluble and ionizable TEL using HME technology. Possible reasons for enhanced solubility and in vitro drug release of the TEL-loaded pH_M_-SD are the microenvironment pH change provided by the addition of an alkalizer that causes enhanced drug release in its favorable pH environment, improved wettability and solubilization with the addition of a hydrophilic polymer, and amorphization of crystalline TEL. Conversely, while the alkalizers can destabilize the amorphous SD system by reducing the T_g_ and increasing moisture adsorption, the stability studies explain that the effect is minimal with HME TEL-loaded pH_M_-SD formulation. Interestingly, the in-house fabricated TEL-SD tablets can withstand the harsh environmental conditions (elevated temp. and moisture) and demonstrated a better stability profile than the commercial tablets. The in vivo pharmacokinetic parameters of the preparations were considerably higher than those of free TEL, primarily due to improved TEL hydrophilicity, which consequently enhanced absorption from the GI tract, resulting in improved oral bioavailability. Collectively, the alkalizer appears to be a promising carrier to fabricate an SD via HME technology, consequently improving in vitro/in vivo profiles and the stability of the system. However, long-term stability studies of the in-house-developed formulation would be required in the future for the practical development of HME TEL-loaded pH_M_ ASD tablets.

## Figures and Tables

**Table 1:** The effect of pH on the solubility of TEL. Each value represents the mean ± standard deviation (*n* = 3).

pH Level	Drug Solubility (µg/mL)
Water	4.42 ± 0.25
pH 1.2	97.18 ± 18.24
pH 4.0	2.46 ± 0.39
pH 6.8	1.26 ± 0.37
pH 10.0	2557.77 ± 171.02

**Table 2:** In vivo pharmacokinetic parameters of TEL powder, TEL-loaded pH_M_-SD, and commercial formulation.

Parameters	TEL	TEL-Loaded pH_M-_ASD	Commercial Formulation
AUC_0_–_∞_ (h·ng/mL)	423.69 ± 114.82	2275.21 ± 776.84 *	3425.42 ± 1553.04 *
C_max_ (ng/mL)	105.46 ± 21.07	697.51 ± 92.65 *	757.27 ± 244.72 *
T_max_ (h)	0.83 ± 0.26	0.75 ± 0.27	1.00 ± 0.55
T_1/2_ (h)	6.62 ± 2.20	4.53 ± 0.974	5.97 ± 1.96
*K*_el_ (h^−1^)	0.11 ± 0.04	0.15 ± 0.04	0.12 ± 0.07

*p < 0.05 compared with free TEL. Each value represents the mean ± SD (*n* = 6).

**Table 3:** Summary of drug and carriers used to develop TEL-loaded pH-modulated solid dispersion.

Structure	Properties	Uses	Ref.
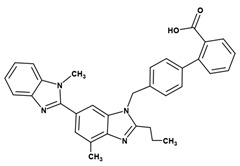 Telmisartan (Mol. Wt. = 514.6 Da)	Weak base, low aqueous solubility (0.09 µg/mL), and hygroscopic in nature	Blood pressure lowering agent (Antihypertensive)	[7]
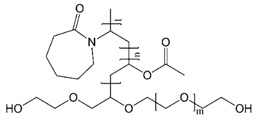 Soluplus^®^ (Mol. Wt. = 90–140 KDa)	Amphiphilic characteristics, low glass transition temperature (T_g_), and low hygroscopicity	Matrix former, solubility enhancer, and stabilizer for SDs	[48,49]
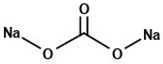 Sodium carbonate (Mol. Wt. = 105.988 Da)	Freely water-soluble white powder with strong alkaline property	Excipients for pharmaceutical formulations	[50]

**Table 4:** Compositions of TEL-loaded solid dispersion (% *w/w*).

Formulations	TEL	SOL	SC	Formulations	TEL	SOL	SC
F1	10	90	0	F7	40	60	0
F2	10	85	5	F8	40	55	5
F3	10	80	10	F9	40	50	10
F4	20	80	0	F10	60	40	0
F5	20	75	5	F11	60	35	5
F6	20	70	10	F12	60	30	10

## Data Availability

The data presented in this study are available in the paper.
